# A Gene-Based Analysis of Acoustic Startle Latency

**DOI:** 10.3389/fpsyt.2017.00117

**Published:** 2017-07-06

**Authors:** Alicia K. Smith, Tanja Jovanovic, Varun Kilaru, Adriana Lori, Lauren Gensler, Samuel S. Lee, Seth Davin Norrholm, Nicholas Massa, Bruce Cuthbert, Bekh Bradley, Kerry J. Ressler, Erica Duncan

**Affiliations:** ^1^Department of Gynecology and Obstetrics, Emory University School of Medicine, Atlanta, GA, United States; ^2^Department of Psychiatry and Behavioral Sciences, Emory University School of Medicine, Atlanta, GA, United States; ^3^Department of Emergency Medicine, University of Texas Southwestern Medical Center, Dallas, TX, United States; ^4^Mental Health Service Line, Atlanta Veterans Affairs Medical Center, Decatur, GA, United States; ^5^Department of Psychiatry, Harvard Medical School, Boston, MA, United States

**Keywords:** acoustic startle, latency, genetics, schizophrenia, endophenotype

## Abstract

Latency of the acoustic startle response is the time required from the presentation of startling auditory stimulus until the startle response is elicited and provides an index of neural processing speed. Latency is prolonged in subjects with schizophrenia compared to controls in some but not all studies and is 68–90% heritable in baseline startle trials. In order to determine the genetic association with latency as a potential inroad into genetically based vulnerability to psychosis, we conducted a gene-based study of latency followed by an independent replication study of significant gene findings with a single-nucleotide polymorphism (SNP)-based analysis of schizophrenia and control subjects. 313 subjects from an urban population of low socioeconomic status with mixed psychiatric diagnoses were included in the gene-based study. Startle testing was conducted using a Biopac M150 system according to our published methods. Genotyping was performed with the Omni-Quad 1M or the Omni Express BeadChip. The replication study was conducted on 154 schizophrenia subjects and 123 psychiatric controls. Genetic analyses were conducted with Illumina Human Omni1-Quad and OmniExpress BeadChips. Twenty-nine SNPs were selected from four genes that were significant in the gene-based analysis and also associated with startle and/or schizophrenia in the literature. Linear regressions on latency were conducted, controlling for age, race, and diagnosis as a dichotomous variable. In the gene-based study, 2,870 genes demonstrated the evidence of association after correction for multiple comparisons (false discovery rate < 0.05). Pathway analysis of these genes revealed enrichment for relevant biological processes including neural transmission (*p* = 0.0029), synaptic transmission (*p* = 0.0032), and neuronal development (*p* = 0.024). The subsequent SNP-based replication analysis revealed a strong association of onset latency with the SNP rs901561 on the neuregulin gene (*NRG1*) in an additive model (beta = 0.21, *p* = 0.001), indicating that subjects with the AA and AG genotypes had slower mean latency than subjects with GG genotype. In conclusion, startle latency, a highly heritable measure that is slowed in schizophrenia, may be a useful biological probe for genetic contributions to psychotic disorders. Our analyses in two independent populations point to a significant prediction of startle latency by genetic variation in *NRG1*.

## Introduction

Schizophrenia is a psychiatric disorder associated with significant morbidity, mortality, and global burden of disease with a prevalence estimated at 1% of the general population (1, 2). Although the cause is multifactorial, the strongest risk factor is family history of the disease, and high heritability up to 80% points to a major role for genetic variation in the etiology of schizophrenia (3). However, schizophrenia does not appear to be inherited in a Mendelian manner and genetic studies of schizophrenia have been complicated by both phenotypic and genetic heterogeneities. Recent genome-wide association studies (GWAS) have made promising steps in identifying independent genomic loci that infer polygenic lifetime risk and heritability to schizophrenia (4–6), but challenges remain in fully deciphering the genetic component of the disease.

One emerging strategy to approach the genetics of schizophrenia is the use of endophenotypes, measurable traits discoverable by a biologic test. To qualify as an endophenotype, the trait must be associated with the disease and be present whether or not the disease is active. The trait must also be heritable, co-segregate with the disease within families, and be found in non-affected family members at a higher rate than the general population (7). The advantage of using endophenotypes to study psychopathology is threefold: first, it reduces the complexity of symptoms and multifaceted behaviors by using quantitative units of measurement; second, a quantitative approach allows more power to detect linkage and is ideal for quantitative trait linkage analysis; third, relatively simpler phenomena involve fewer genes to produce functional abnormalities as compared to psychiatric diagnoses such as schizophrenia. Theoretically, the association between the endophenotype and the genes in question should be stronger than the association to the illness itself when discovered by candidate gene analysis (7, 8).

One endophenotype that is well represented in schizophrenia research is prepulse inhibition of acoustic startle (PPI). The startle reflex, easily measured in animals and humans, is mediated by a simple subcortical circuit (9). PPI is the phenomenon whereby a weaker non-startling “prepulse” stimulus presented shortly before the louder startling stimulus inhibits the magnitude of the startle reflex. PPI has been shown in numerous studies to be impaired in schizophrenia (10).

Another startle measure that has shown promise in recent findings is latency of the acoustic startle response. Startle latency is the time that it takes for the startling stimulus to travel through a three-synapse subcortical circuit that mediates the startle response, thus providing a putative index of neuronal processing speed. The finding of slower acoustic startle latency in schizophrenia than in controls has been reported in a number of studies (10–15), although other papers reported no difference in latency between SCZ and controls (16–27). However, the issue is more nuanced than simple between group differences. Most studies of acoustic startle in schizophrenia used a paradigm that measured PPI. For such sessions, latency facilitation is the characteristic shortening of startle latency in prepulse + pulse trials (in which a non-startling prepulse stimulus precedes the startling stimulus) compared to pulse-alone trials (trials without a prepulse). In some of the studies reporting no overall longer latency in the pulse-alone condition, the schizophrenia subjects had a lesser degree of latency facilitation than controls, thus displaying slower latency on prepulse + pulse trials (16, 22, 27). A heritability analysis by our group found that latency is highly heritable (68–90%) in pulse alone trials and is prolonged both in schizophrenia and family members of subjects with schizophrenia (13).

Importantly for the feasibility of genetic studies, the slowing of latency in subjects with schizophrenia can be detected in those who are treated with antipsychotic medications, as several studies report prolonged latency in schizophrenia cohorts all or most of whom were on antipsychotics (11, 15, 16, 22, 28). Similarly, in two prior published papers on this topic, latency was not affected by medication status (29, 30). However, it should be noted that Weike et al. (27) found differences in latency to prepulse + pulse trials in five unmedicated subjects compared to 20 medicated subjects. Prolonged startle latency is able to identify young subjects at clinical risk for schizophrenia even before they develop schizophrenia (31). Thus, latency shows promise as a potential biomarker of neural abnormalities in schizophrenia and a potential endophenotype. However, a slowing of latency is not unique to schizophrenia. It has been reported in autism (32, 33), Huntington’s disease (34), and post-traumatic stress disorder (25, 35). It is unknown how closely the neural underpinnings of slowed latency in schizophrenia may overlap with that of these other disorders.

Although several endophenotypes have thus far yielded positive findings in genetic association studies with schizophrenia (36), there have been very few studies that have looked at startle latency in relation to genetics relevant to schizophrenia. These studies have focused on single-nucleotide polymorphisms (SNPs) in the human populations and knockout or knockdown genetics in the animal models. Studies in rodents indicate latency association with the *RELN* gene (37), which gene associates with SCZ in human studies (38). Four sites were associated with latency changes in mouse models, most notably *FABP7* (39). There have been even less work looking at genetic variations in latency in human populations. Roussos et al. (40) report an association of slower latency with the DRD3 Ser9Gly polymorphism on SNP rs6280 in healthy humans. To our knowledge, there has not been a study of the genetics of startle latency in subjects with schizophrenia or psychosis. In this study, we conducted a gene-based analysis of startle latency, followed by a replication sample in which SNPs of significant candidate genes were examined.

## Materials and Methods

### Grady Trauma Project Sample

#### Subjects

The subjects in this cohort were part of a larger investigation of genetic and environmental factors that predict the response to stressful life events in a predominantly African American, urban population of low socioeconomic status (41). This study was carried out in accordance with the recommendations of the Emory University Institutional Review Board with written informed consent from all subjects. All subjects gave their informed consent in accordance with the Declaration of Helsinki. The protocol was approved by the Emory University Institutional Review Board and the Grady Health Systems Research Oversight Committee.

Subjects were recruited from the medical, obstetrics-gynecology, and primary care clinic waiting rooms at Grady Hospital in downtown Atlanta, GA, USA. A diagnosis of previous and current mental illnesses was made using either the Structured Clinical Interview for DSM-IV, Axis-I (SCID-I) (42), or the Mini International Neuropsychiatric Interview (MINI) (43). A history of past or current substance use was collected using the SCID-I, the Kreek–McHugh–Schluger–Kellogg (44), and the drug abuse screening test (45). Substance use disorders were classified by summary dichotomous variables based on combined information from the above listed instruments. Current drug use was coded based on current drug use items from these instruments and also a positive or negative urine toxicology that was collected on a subset of subjects. Subjects were classified as having other psychiatric diagnoses based on a combined variable that took into account both SCID-I and MINI data and classified them in a dichotomous variable coding for a history of a given diagnosis vs. no history of that diagnosis. Subjects were excluded if they had a history of sustained head trauma or seizure disorder, major neurologic or medical illness, or hearing impairment as ascertained by a hearing test conducted with an audiometer (Grason-Stadler, Model GS1710). The criterion for adequate hearing acuity was ability to discern tones in each ear of ≤40 dB [A] at 0.25, 0.5, 1, 2, 4, and 8 kHz.

#### Startle Magnitude and Latency Measurements

Subjects were tested in an acoustic startle session designed to assess startle magnitude and fear potentiation of startle. Subjects were seated in a sound attenuating booth during the test session. The eye blink component of the acoustic startle response was measured by electromyography (EMG) of the right orbicularis oculi muscle recorded with the Biopac MP150 system for Windows (Biopac Systems, Inc., Aero Camino, CA, USA) using methods previously published by our group (46). The EMG signal was recorded from two disposable Ag/Ag-Cl electrodes from Biopac positioned 1 cm below the right pupil and 1 cm below the right lateral canthus. Impedance levels were less than 6 kV for each participant as measured by a Checktrode impedance meter (1089 MKIII; UFI, Morro Bay, CA). The recorded EMG signal was sampled at a frequency of 1,000 Hz and was filtered with low frequency (28 Hz) and high frequency (500 Hz) cutoffs. The resultant data were then rectified and smoothed using the MindWare software (Mindware Technologies, Inc., Gahanna, OH, USA).

The acoustic startle probes were 40 ms 108 dB [A] SPL white noise bursts with near instantaneous rise time presented binaurally through Maico headphones (model TDH-39-P). The session began with four trials of noise alone startle stimuli to assess baseline startle magnitude, from which data on acoustic startle latency were extracted. Startle data obtained later in the session to assess fear potentiation were not used for the current analyses.

Startle magnitude for each trial was the maximum EMG signal occurring between 20 and 120 ms after the startling stimuli. Latency was the time of maximal EMG magnitude after the presentation of the startling stimuli (peak latency). Latency data were considered valid if the startle recorded did not begin before 20 ms after the startle stimulus and the startle magnitude was at least 15 µV.

#### DNA Extraction and Genetic Assays

The subjects provided a saliva or blood sample. DNA was extracted from saliva in Oragene collection vials (DNA Genotek Inc., ON, Canada) using the DNAdvance kit (Beckman Coulter Genomics, Danvers, MA, USA), while DNA from blood was extracted using either the E.Z.N.A. Mag-Bind Blood DNA Kit (Omega Bio-Tek, Inc., Norcross, GA, USA) or the ArchivePure DNA Blood Kit (5 Prime, Inc., Gaithersburg, MD, USA).

Genotyping was performed using the Omni-Quad 1M (Illumina, San Diego, CA, USA). Genotypes were called using the Illumina’s GenomeStudio software. We used PLINK to perform quality-control (QC) analyses on the genetic data. In brief, initial QC involved removing samples with very low call rates and those outside acceptable levels of heterozygosity (−0.25 < Fhet > 0.25); the remaining samples were recalled in GenomeStudio. We then removed SNPs with call rates less than or equal to 98%, a frequency of less than 0.01, and individuals with greater than 2% missing data. We further identified and removed related individuals by using PLINK to estimate the proportion of identity by descent (IBD) for each pair of individuals. Among pairs of individuals with an IBD proportion >0.12 (indicating cousins or a closer relation), we removed the individual in each pair with the higher rate of missing genotype data. Using data (autosomes only) pruned in PLINK, we performed principal-component analysis to infer axes of ancestry. Subjects that fell within 3 SDs of the medians of the first and second PCs in our sample were considered outliers and removed.

#### Statistical Analysis

PLINK was used to regress startle latency on allele count assuming an additive model (0, 1, or 2 copies of a risk allele), including sex, and the top 10 PCs of genome-wide data as covariates (47, 48). Gene-based association tests were performed using the minSNP method as implemented in FAST using the summary data derived from the SNP-based association tests described above (49). The gene-based *p*-value was calculated by using the permuted *p*-value of the best individual SNP association within the given gene. minSNP computes single SNP *F*-statistics for each SNP within a gene and uses the best *F*-statistic within that gene as its test statistic. The *p*-value was calculated using 1,000,000 permutations to correct for gene size (50). The precomputed haplotype and index files for the ASW population were downloaded from the FAST wiki page and used as a reference. The number of tests performed was calculated based on the number of genes analyzed (*N* = 32,194), and the false discovery rate (FDR) was controlled at 5% to account for multiple testing. Pathway analysis was performed with DAVID (51), and the FDR was controlled at 5%.

### Independent Replication Sample of Veterans Affairs Subjects

#### Subjects

We next focused on these genes in a SNP-based replication study on the Atlanta Veterans Affairs Medical Center (VA) cohort, which consisted of 324 subjects, of whom 185 had schizophrenia and 139 were psychiatric controls. This study was carried out in accordance with the recommendations of the Emory University Institutional Review Board with written informed consent from all subjects. All subjects gave their informed consent in accordance with the Declaration of Helsinki. The protocol was approved by the Emory University Institutional Review Board and the Atlanta Veterans Affairs Medical Center Human Subjects Committee.

We examined regressions on both onset latency (the time from presentation of the startling stimulus until the onset of the startle blink) and peak latency (the time from presentation of the startling stimulus until the time of peak magnitude of the startle blink). The subjects in this independent replication sample were collected through a VA funded Merit Review Project [PI, (29)]. The sample consisted of 185 unrelated schizophrenia subjects (SCZ) and 139 psychiatric control subjects (CON) with DNA extracted from blood samples and startle testing completed. Demographic data were collected on all subjects, and psychiatric medication status was collected on the SCZ group. Current symptom ratings were conducted on the SCZ subjects by means of the Positive and Negative Syndrome Scale (52). The DSM-IV diagnosis for SCZ subjects or lack thereof for CON subjects was confirmed based on the SCID-I (42). Female subjects were tested during the first 2 weeks of their menstrual cycle (follicular phase), because prior work indicates that women express a reduced prepulse inhibition during the luteal phase (53, 54).

#### Startle Magnitude and Latency Measurements

Hearing acuity was assessed by means of audiometer testing with a Grason-Stadler audiometer (Model GS1710). To be included, subjects had to detect tones in each ear at a threshold of 40 dB [A] at 0.25, 0.50, 1, 2, 4, and 8 kHz. Subjects completed a startle paradigm designed to assess magnitude, latency, and prepulse inhibition of startle using methods developed by Braff and colleagues (17) as described in prior publications from our group (13, 55). Briefly, subjects were seated in a sound attenuating booth and asked to look straight ahead and keep their eyes open during the testing. Acoustic stimuli were delivered binaurally through headphones (Maico, TDH-39-P). The eye blink component of the acoustic startle response was measured *via* EMG recording of the right *orbicularis oculi* muscle. Two Ag/Ag-Cl electrodes were positioned 1 cm below and right pupil and 1 cm below the right lateral canthus, with a ground electrode behind the right ear. The resistances for all subjects were less than 6 kΩ as measured with a Checktrode impedance meter (1089 MKIII; UFI, Morro Bay, CA, USA). EMG activity was amplified and digitized using a computerized EMG startle response monitoring system (SR-LAB, San Diego Instruments). The EMG signal was filtered with low- and high-frequency cutoffs at 30 and 1,000 Hz, respectively. The system was set to record 250 1-ms readings starting at the onset of the startle (pulse alone) stimulus. Digital signals were full-wave rectified and smoothed by an averaging routine that calculates a rolling average of 10 data points by means of SR-LAB analysis software.

The startle session began with a 60-s acclimation period consisting of 70 dB white noise that continued as background noise throughout the session. The pulse-alone stimuli were 116 dB, 40-ms bursts of white noise. The first block of the session consisted of six pulse alone stimuli. From this block, data on startle magnitude and latency were extracted for the current analyses. The next portion of the session, designed to assess prepulse inhibition of acoustic startle, was not used in the analyses presented herein.

#### DNA Extraction and Genetic Assays

To select genes for the focused replication analysis, we filtered the 2,870 significant genes from the gene-based analysis above based on whether they are expressed in the pons, a region of the brain that mediates startle response, and whether they have a prior association in the literature with startle (either latency or magnitude) or with schizophrenia or schizophrenia-related endophenotypes. This resulted in the following four genes that met these criteria: disrupted in schizophrenia 1 (*DISC1*), v-erb-b2 avian erythroblastic leukemia viral oncogene homolog (*ERBB4*), nitric oxide synthase 1 (neuronal) adapter protein (*NOS1AP*), and neuregulin (*NRG1*).

We proceeded with a SNP-based analysis of these four genes. Genotyping was performed in 384-well format using iPlex Gold kits and the Sequenom MassARRAY system. Amplification and extension primers were designed by SpectroDESIGNER software. The MassARRAY™ Typer software was used to assign the genotype calls. Each 384-well genotyping plate contained positive and negative controls. No samples were excluded based on missing data rates >5%. In total, 38 SNPs were genotyped of which 9 were eliminated because of call rates <95% and deviations from Hardy–Weinberg proportions (*p* ≤ 0.01), resulting in 29 SNPs that were analyzed for their relation with latency. Six SNPs had minor allele frequencies below 5%, and MAF was considered in interpretation of the results.

#### Statistical Analysis

We examined the association between latency and each SNP under one of the following two inheritance models: additive and dominant. For the additive model, each genotype was coded numerically, such that latency levels of heterozygotes should fall in between both homozygous groups. For those SNPs having a cell size for the minor allele less than 5, dominant models were evaluated assuming that a single copy of an allele is sufficient to influence the trait. We examined regressions on both onset latency (the time from presentation of the startling stimulus until the onset of the startle blink) and peak latency (the time from presentation of the startling stimulus until the time of peak magnitude of the startle blink). For each of these potential models, we examined association using linear regressions that incorporated covariates of age, race, and diagnosis (SCZ vs. CON). All *p*-values reported are two-sided.

## Results

### Gene-Based Analysis of Grady Sample

Demographic information on the Grady sample is listed in Table 1. Of the 380 subjects tested, 19 subjects were non-startlers and, therefore, did not have valid latency measures. An additional 48 were removed during GWAS QC. Therefore, the final analyses were conducted on 313 subjects. The mean age was 41.1 ± 12.1 years. 69.3% of the subjects were female and 30.7% were male. The population was primarily African American at 98.7%, with 1.3% being Caucasian. Additional diagnostic information and latency values for subgroups of subjects are presented in Table 1. No diagnostic subgroups had significant differences in peak latency by *t*-tests, as is noted in Table 1. Urine toxicology testing was available on a subset of Grady subjects (*n* = 48). Latency did not differ between those who were positive vs. negative (*t* = −0.87, *p* = 0.40). Additionally, those subjects admitting to current drug use on structured interview or having positive urine toxicology at the time of testing did not differ significantly in latency from those without current drug use or positive urines (*t* = −0.994, *p* = 0.32).

**Table 1:** Demographic and clinical characteristics of the final Grady sample (*N* = 313).

Characteristic	Mean or percent	Peak latency^a^
Age (years, mean ± SD)	41.1 ± 12.1	71.7 ± 24.6
Sex, *N* (%)		
Male	30.7	72.1 ± 23.4
Female	69.3	71.6 ± 25.2
Race, *N* (%)		
African American	98.7	71.8 ± 24.8
Caucasian	1.3	62.1 ± 11.2
Controls (no psychiatric diagnosis), %		
No	85.1	73.0 ± 25.3
Yes (i.e., controls)	14.9	66.8 ± 23.5
History of schizophrenia, %		
No	91.9	71.6 ± 25.3
Yes	8.1	71.2 ± 14.1
History of bipolar disorder, %		
No	66.7	72.0 ± 25.9
Yes	33.3	78.7 ± 24.0
History of major depression, %		
No	50.6	71.4 ± 27.7
Yes	49.4	73.2 ± 24.0
History of PTSD, %		
No	44.4	70.1 ± 23.4
Yes	55.6	73.0 ± 25.6
History of alcohol use disorder, %		
No	56.6	72.3 ± 26.1
Yes	43.4	72.2 ± 25.7
History of any drug use disorder, %		
No	44.4	74.3 ± 30.5
Yes	55.6	70.7 ± 20.8
History of cocaine use disorder, %		
No	70.2	73.4 ± 28.1
Yes	29.8	68.3 ± 17.3

*^a^Latency differences between all subgroups by t-test: p > 0.05*.

**Figure 1:**
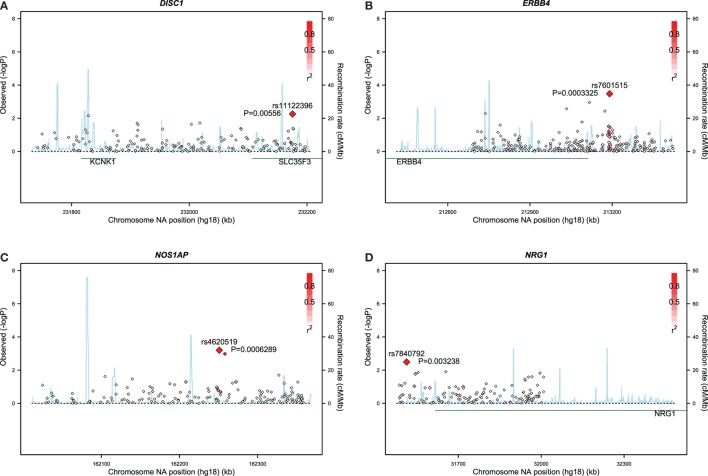
Results of gene-based analyses of the Grady sample for: **(A)** *DISC1*, **(B)** *ERBB4*, **(C)** *NOS1AP*, and **(D)** *NRG1*.

Gene-based associations for startle latency are summarized in Table 2. Consistent with the high degree of heritability for startle latency, 2,870 genes demonstrated evidence of association after correction for multiple comparisons (FDR < 0.05; Table S1 in Supplementary Material). Pathway analysis of these genes revealed enrichment for relevant biological processes including transmission of nerve impulses (*p* = 0.0029), synaptic transmission (*p* = 0.0032), and neuron development (*p* = 0.024) among others (Table 2). Figure 1 shows details of the gene-based analysis for four genes that were significant in this analysis after Bonferroni correction for multiple comparisons, are expressed in the pons, and known to be associated with schizophrenia: *DISC1, ERBB4, NOS1AP*, and *NRG1*.

**Table 2:** Biological processes enriched in pathway analysis of significant gene-based associations with startle latency.

Gene function	GO number	Genes	Enrichment score	*p*-Value	Corrected *p*-value
Biological adhesion	GO:0022610	135	5.4	4.30E−12	8.10E−09
Cell adhesion	GO:0007155	135	5.4	3.90E−12	1.50E−08
Transmission of nerve impulse	GO:0019226	67	2.7	2.30E−06	2.90E−03
Synaptic transmission	GO:0007268	59	2.4	3.40E−06	3.20E−03
Cell projection organization	GO:0030030	67	2.7	1.30E−05	9.80E−03
Cell morphogenesis	GO:0000902	65	2.6	1.60E−05	1.00E−02
Cellular component morphogenesis	GO:0032989	70	2.8	2.40E−05	1.30E−02
Calcium ion transport	GO:0006816	33	1.3	2.80E−05	1.30E−02
Synapse organization	GO:0050808	19	0.8	4.00E−05	1.70E−02
Calcium-dependent cell–cell adhesion	GO:0016339	11	0.4	5.50E−05	1.90E−02
Cell–cell adhesion	GO:0016337	52	2.1	5.50E−05	2.10E−02
Neuron development	GO:0048666	60	2.4	8.90E−05	2.40E−02
Cell morphogenesis involved in differentiation	GO:0000904	47	1.9	7.60E−05	2.40E−02
Proteoglycan metabolic process	GO:0006029	15	0.6	8.80E−05	2.50E−02
Cell recognition	GO:0008037	17	0.7	1.30E−04	3.00E−02
Neuron projection development	GO:0031175	48	1.9	1.20E−04	3.10E−02

### Replication Study of Veterans Affairs Subjects

Table 3 displays demographic and clinical characteristics of this sample consisting of 185 subjects with schizophrenia and 139 psychiatric controls. Of this sample, 63 subjects were non-startlers and, therefore, did not have evaluable latency measures. Therefore, the final analyses were conducted on 277 subjects. This sample was more racially diverse and contained a greater percentage of males than the GTP sample. The SCZ sample consisted predominantly of outpatients stabilized on antipsychotic medication, although 15 subjects were unmedicated at the time of testing.

**Table 3:** Demographic and clinical characteristics of the final VA sample.

Charactieristic	CONT (*n* = 123)	SCZ (*n* = 154)
Age (years, mean ± SD)^a^	36.6 ± 14.0	44.4 ± 10.4
Sex (%)^b^		
Female	62 (50.4)	31 (20.1)
Male	61 (49.6)	123 (79.9)
Race (%)^c^		
African American or others	65 (52.8)	95 (61.7)
Caucasian	58 (47.2)	59 (38.3)
Positive and Negative Syndrome Scale rating (mean ± SD)		
Total	–	62.5 ± 16.8
Positive symptoms	–	17.2 ± 5.7
Negative symptoms	–	15.5 ± 6.2
General psychopathology	–	30.0 ± 8.6

*CONT, control subjects; SCZ, subjects with schizophrenia*.

*^a^Age CON and SCZ (*t*-test): *t* = −5.30, *p* < 0.001*.

*^b^Sex between groups (Chi-square): *X*^2^ = 28.11, df = 1, *p* < 0.001*.

*^c^Race between groups (Chi-square): *X*^2^ = 2.19, df = 1, *p* = 0.14*.

Table 4 shows the onset latency and peak latency values for this independent follow-up sample. Neither onset latency nor peak latency differed in SCZ or CON subjects according to sex. Regarding race, in CON only, the African American or other subjects had longer onset latency than Caucasian subjects. Onset latency and peak latency were significantly correlated in the VA sample as a whole (Pearson *r* = 0.560, *p* < 0.001), in the SCZ subjects (Pearson *r* = 0.56, *p* < 0.001), and in the CON subjects (Pearson *r* = 0.57, *p* < 0.001).

**Table 4:** Latency values for the VA sample.

	Onset latency^a^		Peak latency^a^	
Characteristic	CONT	SCZ	CONT	SCZ
Sex				
Female	51.5 ± 16.7	53.0 ± 16.7	81.4 ± 18.4	89.2 ± 15.9
Male	54.1 ± 14.3	55.7 ± 15.9	81.3 ± 17.7	84.6 ± 18.7
Race^b^				
African American or others	56.4 ± 16.2	56.0 ± 16.3	84.2 ± 17.7	84.7 ± 19.2
Caucasian	48.7 ± 13.9	53.9 ± 15.7	78.1 ± 17.9	86.8 ± 16.6

*CONT, control subjects; SCZ, subjects with schizophrenia*.

*^a^Values are mean ± SD*.

*^b^t-test for race, African American or others > Caucasian: p < 0.006*.

**Figure 2:**
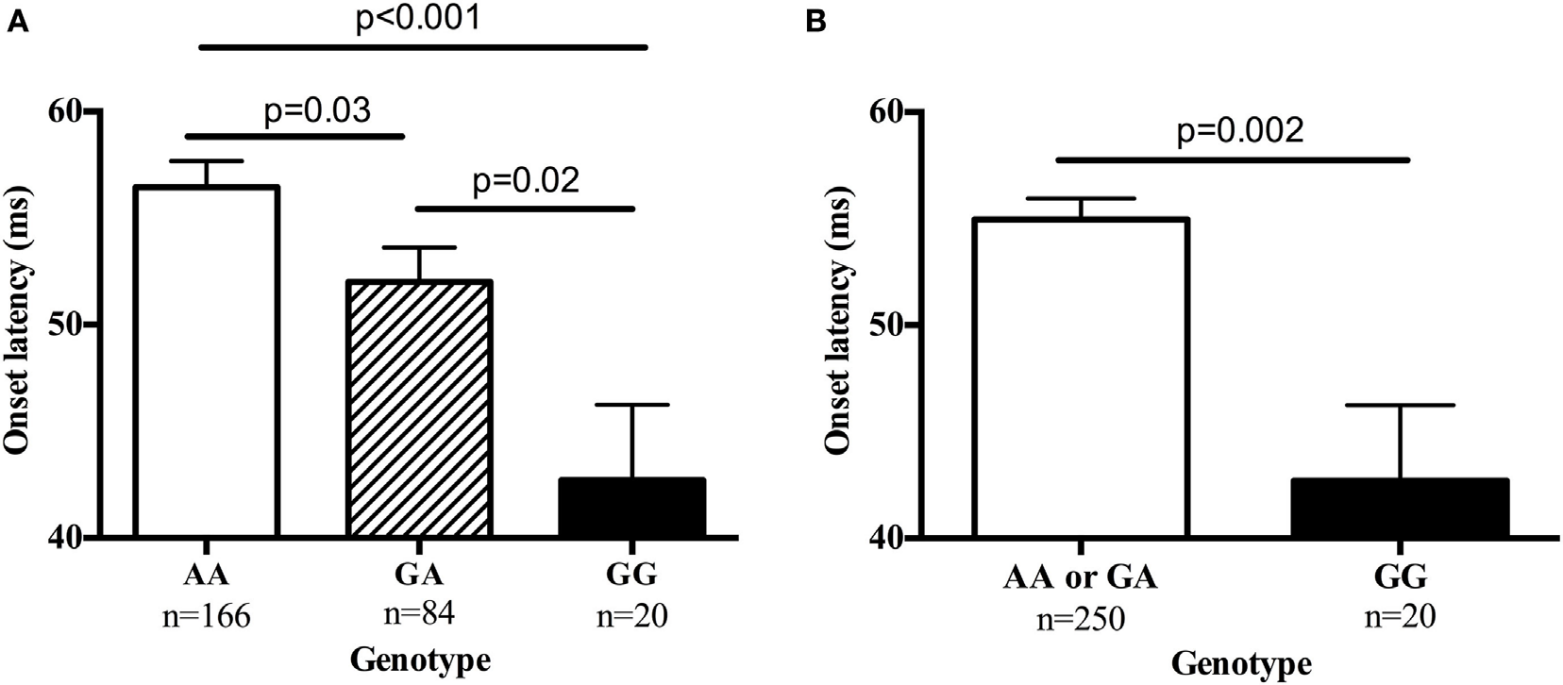
The single-nucleotide polymorphism rs901561 on the neuregulin gene (*NRG1*) predicted onset latency. Values are mean ± SEM. **(A)** Results of additive model (beta = 0.21, *p* = 0.001). *p*-Values shown are for post hocs. **(B)** Results of dominant model (beta = −0.15, *p* = 0.02). *p*-Value shown is for one-way ANOVA.

Table S2 in Supplementary Material provides details for the 29 SNPs that were tested on these four genes. Table 5 summarizes results for SNPs on the four genes selected for our independent follow-up study (*DISC1, ERBB4, NOS1AP*, and *NRG1*) that predicted slowing of latency (*p* < 0.05) in either an additive or dominant models. After Bonferroni correction of *p* = 0.0017 for multiple comparisons, only the finding for the *NRG1* gene was significantly associated with startle latency. Our analysis revealed a strong association of onset latency with SNP rs901561 on *NRG1*, as can be seen in additive and dominant models shown in Figure 2. Subjects with the AA and AG genotypes of rs901561 (*NRG1*) had slower mean latency than subjects with the GG genotype (beta = 0.21, *p* = 0.001).

**Table 5:** Association of single-nucleotide polymorphisms (SNPs) with startle latency^a^.

			Association with onset latency		Association with peak latency	
Gene	SNP	Location	Beta	*p*-Value	Beta	*p*-Value
*DISC1*	rs2082552	1q42.1; INTRON	0.10	0.12	0.14	0.03
*DISC1*	rs3738402	1q42.1; INTRON, synonymous codon	0.08	0.19	0.16	0.01^b^
*ERBB4*	rs10932374	2q33.3–q34; UTR	−0.12	0.05	−0.04	0.49
*ERBB4*	rs12467225	2q33.3–q34; UTR	0.12	0.05	0.07	0.26
*NRG1*	rs901561	8p12; INTRON	0.21	0.001	0.03	0.67

*^a^Regressions included age, race, and diagnosis (schizophrenia vs. control). Additive models were used unless minor allele frequency was <5*.

*^b^Dominant model used because minor allele frequency was <5*.

Two SNPs in *DISC1* predicted slowing of latency, although the significance level did not survive our correction for multiple comparisons. Subjects with the AA genotype at rs2082552 had slower predicted latency than subjects with GG, with AG subjects intermediate (beta = 0.14, *p* = 0.03). The *DISC1* SNP, rs3738402, significantly predicted peak latency in a dominant model (beta = 0.16, *p* = 0.01), with subjects with the CT or TT genotypes demonstrating slower latency than those with the CC genotype. For this SNP, there was only one TT homozygote, so an additive model could not be computed.

Two SNPs on *ERBB4* also predicted latency, although the significance level did not survive our correction for multiple comparisons. Subjects with the GG genotype at rs10932374 had slower predicted onset latency than AA subjects, with AG subjects having intermediate latency (beta = −0.12; *p* = 0.05). Similarly, rs12467225 also predicted onset latency in an additive model (beta = 0.12; *p* = 0.05), with subjects that had the CC genotype exhibiting slower latency than TT subjects and CT subjects exhibiting intermediate latency.

## Discussion

In this study, we used a gene-based analysis to examine the association of startle latency with multiple genes in a hypothesis neutral manner. This revealed associations with 2,870 genes involved in neural transmission, synaptic transmission, and neuronal development. We then conducted a SNP-based follow-up study on an independent sample targeting genes expressed in the pons, a region that mediates startle, and known from prior work to be associated with schizophrenia, schizophrenia endophenotypes, or acoustic startle. This SNP-based analysis revealed a significant association of startle latency with a SNP on the *NRG1* gene, with two loci on the *DISC1* gene, and with two loci on the *ERBB4* gene, although these latter four loci did not survive Bonferroni correction.

The gene-based analysis revealed a significant association of >2,000 genes with startle latency. The preponderance of these genes is known to be involved in processes that could be expected to be relevant to startle latency, such as transmission of nerve impulses, synaptic transmission, and neuronal development. We have hypothesized that slowing of startle latency may reflect a more generalized slowing of neural transmission (13). Although it is currently not known which processes that subserve neural transmission may be affected in schizophrenia such that these individuals have slowed latency, the genetic findings that we report herein provide clues to the potential molecular underpinnings of slowed latency in schizophrenia.

Our analysis revealed a strong association of latency with the *NRG1* gene in our gene-based analysis, and in our SNP-based replication, rs901561 was significant in both additive and dominant models. This gene has been reported as being associated with SCZ in large GWAS studies (56, 57) and confirmed by meta-analysis (58, 59), but the association with slowing of latency suggests that this SNP may identify a subset of individuals with the biomarker of slowed latency. It should be noted, however, that a prior study did not detect a significant association of a different SNP with startle latency in healthy controls (60). The gene is expressed in the pons (61), although the relationship between rs901561 and *NRG1* remains to be determined. Neuregulins have frequently been associated with schizophrenia, as those deficient in *NRG1* have fewer working NMDA receptors. NMDA receptors directly aid in glutamate transmission, and disruptions in glutamate signaling have been widely linked to schizophrenia (36, 56, 62). In the Greenwood et al. study (36, 63), *NRG1* was significantly associated with several of their endophenotypes, including startle magnitude, prepulse inhibition of startle, verbal learning, abstraction, spatial processing, and sensorimotor dexterity. It may be that cognitive and motor performance impairments associated with *NRG1* genotypes are in part mediated or modulated by slowing of neural processing as indicated by slowed startle latency. We have speculated that slowing of neural processing could lead to the dysfunction of neural circuits subserving cognitive or psychomotor performance, although to date the two studies examining startle latency and cognition have not found significant correlations (15, 64). However, a preliminary analysis of data from our lab has found a significant association between onset latency and cognitive function after adjusting for sex, race, age, and diagnosis (SCZ vs. CON; *N* = 221). In the Finger Tapping Task, a test of motor speed, we found that a higher score (i.e., more taps in a set period of time) corresponded with faster onset latency in both dominant (*B* = −0.038, *p* = 0.048) and non-dominant (*B* = −0.057, *p* = 0.014; unpublished data) hands.

It is worth noting the association of two *ERBB4* SNPs with latency, rs10932374 and rs12467225, although the significance for these SNPs did not survive correction for multiple comparisons. *ERBB4* has been extensively investigated as a vulnerability gene for schizophrenia (65–67). It was included in the Consortium on Schizophrenia study and yielded significance for several endophenotypes, notably P50 gating, antisaccades, and several cognitive measures (36, 63). Neuregulin signaling *via* ERBB4 receptors has an important role in axonal guidance (68) and control of activity-dependent dendritic spines (69). The neuregulin-ERBB4 signaling pathway is emerging as being important in both glutamatergic and GABAergic neurotransmissions (70) [see review in Ref. (71)], and both these neurotransmitters are robustly implicated in the pathogenesis of schizophrenia (62, 72). The fact that our analysis revealed associations of latency with both *NRG1* and *ERBB4* renders these finding complementary and suggests an involvement of the neuregulin-ERRB4 signaling pathway as a potential route by which slowed latency, and neural slowing, could emerge in genetically vulnerable individuals.

It is also worth noting that our SNP-based analysis revealed significant associations of latency with two SNPs on the *DISC1* gene, rs2082552 and rs3738402, although these associations were above the level of significance required by a Bonferroni correction. *DISC1* is a known vulnerability gene for schizophrenia (73–77). Several lines of evidence implicate the role of the *DISC1* gene in the etiopathophysiology of SCZ (78, 79). The gene encodes a protein that modulates cortical growth and development, which could explain why mutations and malfunctions in the gene could increase latency and slow neuronal processing speed. *DISC1* variants are associated with startle magnitude (63), P300 deficits (73, 80), sensory gating deficits, antisaccade deficits (36), and cognitive deficits (36, 78). In parallel with our current findings, prolonged latency of the P300 wave was associated with a *DISC1* locus translocation (73), and the authors postulate that this P300 prolongation indicates slowed neural processing of the stimuli.

Our genetic findings for onset latency and peak latency in the VA sample were divergent, even though there was a robust correlation between onset latency and peak latency for the sample as a whole and for each subject group separately. The analyses on the Grady sample used peak latency. In the literature regarding latency differences between SCZ and CON, onset only was used in one study (27), peak was used in six studies (10, 12, 14–16, 19), and both onset and peak were used in three studies (11, 13, 22). The reason that these two closely correlated measures yield different results is unclear at this time.

One strength of this study is the use of a gene-based analysis for discovery, with replication in an independent sample. Genetic associations can be difficult to replicate between cohorts due to differences in power, genetic ancestry, or clinical factors. Gene-based associations are more likely to replicate across populations because they aggregate signals across functional units of gene expression and have a lower threshold for multiple test correction (81, 82). Gene-based association tests complement traditional genetic approaches and have been used to identify additional genes associated with complex traits in previous studies (83–86).

The study has several limitations. First, the study size limited the ability to detect significance when correcting for multiple tests. A Bonferroni correction for testing of 29 SNPs yields a corrected *p*-value of 0.0017, which significance threshold was not reached by several of our significant findings. A larger sample size would be ideal to adequately detect significance in many of the SNPs that were trending toward corrected significance. Additionally, because of the smaller sample size, many of the findings in the additive models could not be interpreted since the least frequent genotype was too small to be able to interpret the results. Second, in the SNP-based replication study, we looked at only a limited number of SNPs on the four genes that emerged from our gene-based analysis. It is possible that had we analyzed additional SNPs on these genes, additional significant results would have emerged. Third, the gene-based part of this study looked at a predominantly African American population and, thus, the results may not be generalizable to other populations. However, our replication study in the VA population consisted of a sample that was quite well balanced racially. Fourth, a sizable percent of the Grady subjects had current or past substance use disorders and 49 subjects either admitted to current drug use or had positive urine toxicologies. Latency was not significantly different in this currently using group than those without current drug use, but our findings would have been strengthened had urine testing been available on all Grady subjects.

In summary, the results of this study need replication in a larger data set, but the preliminary results support an association of startle latency with the multiple genes involved in neuronal signaling and development, including the *NRG1* gene. A significant association of latency with a SNP on *NRG1* was found in an independent subject sample. Evidence of longer (i.e., slower) acoustic startle latency as indirect evidence of slowing of neural processing supports the underlying hypothesis that genetically determined alterations of the neuregulin-ERBB4 and glutamate signaling pathways play a role in the pathophysiology of schizophrenia. These results need replication in larger datasets, and future studies should be conducted on other genes that were significant in our discovery sample. However, our latency findings may provide a foundation from which we can characterize a distinct subgroup of schizophrenia patients and discover the neurobiology underlying the association of slowed latency with schizophrenia risk.

## Ethics Statement

This study was carried out in accordance with the recommendations of the Emory University Institutional Review Board with written informed consent from all subjects. All subjects gave their informed consent in accordance with the Declaration of Helsinki. The protocol was approved by the Emory University Institutional Review Board.

## Author Contributions

AS, TJ, KR, and ED designed the experiments. AS, TJ, SN, BC, BB, KR, and ED performed the experiments. AS, TJ, VK, AL, LG, SL, NM, BB, KR, and ED analyzed the data. AS, LG, SL, and ED wrote the manuscript. All authors edited and approved the manuscript.

## Conflict of Interest Statement

The authors declare that the research was conducted in the absence of any commercial or financial relationships that could be construed as a potential conflict of interest.
