# Supplementary material for: A Gene-Based Analysis of Acoustic Startle Latency
Source: Front Psychiatry. 2017 Jul 6;8:117. doi: 10.3389/fpsyt.2017.00117 (PMC5498475; doi:10.3389/fpsyt.2017.00117)
Supplement: Supplementary file 3 [file table_3.pdf]

Author, Year	Journal	Subject groups	Latency type	Overall between group latency findings	Latency facilitation findings (latency in prepulse trials)
Weike et al. 2000	Biol Psychiatry	20 SCZ medicated; 5 SCZ unmedicated; 12 CON	ONSET	SCZ=CON	Unmedicated SCZ>CON in 30ms prepulse trials
Braff et al. 1992	Arch Gen Psychiatry	39 SCZ; 37 CON	ONSET & PEAK	SCZ=CON	SCZ=CON
Braff et al. 1999	Am J Psychiatry	51 SCZ; 26 CON	ONSET & PEAK	SCZ>CON in onset and peak	SCZ=CON
Hasenkamp et al. 2010	Psych Res	107 SCZ, 94 CON	ONSET & PEAK	SCZ>CON in onset and peak	SCZ>CON in 30ms, 60 ms, 120 ms prepulse trials
Kumari et al. 2000	Arch Gen Psychiatry	29 SCZ on atypicals; 9 SCZ on typicals; 20 CON	ONSET & PEAK	SCZ=CON	
Mackeprang et al. 2002	Biol Psychiatry	20 SCZ; 20 CON	ONSET & PEAK	SCZ=CON	SCZ=CON
Parwani et al. 2000	Biol Psychiatry	24 SCZ; 20 CON	ONSET & PEAK	SCZ=CON	SCZ=CON
Braff et al. 1978	Psychophysiol	12 SCZ; 20 CON	PEAK	SCZ=CON	SCZ>CON in 60ms prepulse trials
Cadenhead et al. 2000	Am J Psychiatry	23 SCZ; 34 family; 11 schizotypals; 25 CON	PEAK	SCZ=CON	SCZ=CON
Geyer and Braff 1982	Psychophysiol	22 SCZ; 20 other diagnoses; 25 CON	PEAK	SCZ=CON	n.a.; PPI session not done
Leumann et al. 2002	Biol Psychiatry	17 SCZ on atypicals; 16 SCZ on typicals; 19 CON	PEAK	SCZ=CON	SCZ=CON
Ludewig et al. 2003	Biol Psychiatry	24 SCZ; 21 CON	PEAK	SCZ=CON	SCZ=CON
Ludewig et at. 2002	Schizophr Res	19 SCZ; 24 CON	PEAK	SCZ=CON	SCZ>CON in 30ms and 60 ms prepulse trials
Swerdlow et al. 2006	Arch Gen Psychiatry	103 SCZ; 66 CON	PEAK	SCZ>CON	SCZ=CON
Braff et al. 2001	Schizophr Res	31 SCZ; 34 CONTROLS	PEAK	SCZ>CON	SCZ=CON
Cosmor et al. 2009	Schizophrenia Bull	24 SCZ medicated; 13 SCZ unmedicated; 43 CON	PEAK	SCZ>CON	SCZ>CON in 120ms prepulse trials
Storozheva et al. 2016	Spanish J Psychology	28 SCZ; 25 CON	PEAK	SCZ>CON	not reported
